# Vision-language model performance on the Japanese Nuclear Medicine Board Examination: high accuracy in text but challenges with image interpretation

**DOI:** 10.1007/s12149-025-02084-x

**Published:** 2025-07-15

**Authors:** Rintaro Ito, Keita Kato, Marina Higashi, Yumi Abe, Ryogo Minamimoto, Katsuhiko Kato, Toshiaki Taoka, Shinji Naganawa

**Affiliations:** 1 Department of Radiology, Nagoya University Graduate School of Medicine, Nagoya, Aichi Japan; 2 Department of Innovative BioMedical Visualization (iBMV), Nagoya University Graduate School of Medicine, 65, Tsurumaicho, Shouwa-Ku, Nagoya, Aichi 466-8550 Japan; 3 Department of Integrated Image Information Analysis, Nagoya University Graduate School of Medicine, Nagoya, Aichi Japan; 4 Functional Medical Imaging, Biomedical Imaging Sciences, Division of Advanced Information Health Sciences, Department of Integrated Health Sciences, Nagoya University Graduate School of Medicine, Nagoya, Aichi Japan

**Keywords:** Artificial intelligence, Large language models, Vision language models

## Abstract

**Objective:**

Vision language models (VLMs) allow visual input to Large Language Models. VLMs have been developing rapidly, and their accuracy is improving rapidly. Their performance in nuclear medicine compared to state-of-the-art models, including reasoning models, is not yet clear. We evaluated state-of-the-art VLMs using problems from the past Japan Nuclear Medicine Board Examination (JNMBE) and assessed their strengths and limitations.

**Methods:**

We collected 180 multiple-choice questions from JNMBE (2022–2024). About one-third included diagnostic images. We used eight latest VLMs. ChatGPT o1 pro, ChatGPT o1, ChatGPT o3-mini, ChatGPT-4.5, Claude 3.7, Gemini 2.0 Flash thinking, Llama 3.2, and Gemma 3 were tested. Each model answered every question three times in a deterministic setting, and the final answer was set by majority vote. Two board-certified nuclear medicine physicians independently provided reference answers, with a third expert resolving disagreements. We calculated overall accuracy with 95% confidence intervals and performed subgroup analyses by question type, content, and exam year.

**Results:**

Overall accuracies ranged from 36.1% (Gemma 3) to 83.3% (ChatGPT o1 pro). ChatGPT o1 pro achieved the highest score (150/180, 83.3% [95% CI: 77.1–88.5%]), followed by ChatGPT o3-mini (82.8%) and ChatGPTo1 (78.9%). All models performed better on text-only questions than on image-based ones; ChatGPT o1 pro correctly answered 89.5% of text questions versus 66.0% of image questions. VLMs demonstrated limitations in handling with questions on Japanese regulations. ChatGPT 4.5 excelled in neurology-related image-based questions (76.9%). Accuracy was slightly lower from 2022 to 2024 for most models.

**Conclusions:**

VLMs demonstrated high accuracy on the JNMBE, especially on text-based questions, but exhibited limitations with image recognition questions. These findings show that VLMs can be a good assistant for text-based questions in medical domains but have limitations when it comes to comprehensive questions that include images. Currently, VLMs cannot replace comprehensive training and expert interpretation. Because VLMs evolve rapidly and exam difficulty varies annually, these findings should be interpreted in that context.

## Introduction

The latter half of 2024 and early 2025 witnessed a significant proliferation of new Large Language Models (LLMs). Notably, within the commercial sector, a succession of reasoning models was introduced by OpenAI, beginning with ChatGPT o1 pro and ChatGPT o1 in December 2024, followed by ChatGPT o3-mini in January 2025 [1, 2]. While not a reasoning model, OpenAI also released ChatGPT-4.5 on February 27, 2025, which provided updates to their existing models [3]. Anthropic’s Claude 3.7 Sonnet, launched in February 2025, incorporated reasoning capabilities, representing an evolution from its previous iterations to reasoning-based architecture [4]. Similarly, Google DeepMind entered this space with Gemini 2.0 Flash thinking in February 2025, another reasoning model [5]. This marked transition of numerous cloud-based models toward reasoning frameworks has reportedly enhanced overall LLM performance. Concurrently, developments in local models occurred with Meta's Llama 3.2, released on September 25, 2024, which featured vision capabilities, and Google DeepMind's Gemma 3, introduced on March 12, 2025, a multimodal model [6, 7]. While these local models do not incorporate reasoning mechanisms, they offer the distinct advantage of being executable on personal computing devices.

The field of nuclear medicine relies heavily on the interpretation of complex medical images alongside clinical knowledge for accurate diagnosis and treatment. The Japanese Nuclear Medicine Board Examination (JNMBE) serves as a critical benchmark for ensuring the competence of specialists in this domain. In recent years, LLMs have shown remarkable progress in various fields, including medicine, demonstrating potential in tasks ranging from answering medical questions to clinical reasoning [8–11]. The advent of vision-language models (VLMs), which can process both textual and visual information, has opened new avenues for their application in specialties like radiology and nuclear medicine, where image analysis is fundamental [12, 13].

Several studies have begun to explore the capabilities of LLMs and VLMs in the context of medical board examinations. Other research has focused on LLMs' ability to process and interpret medical reports in specific contexts, such as classifying lymphoma in 18 F-fluorodeoxyglucose-positron emission tomography (FDG-PET) reports based on the Lugano criteria [14]. Understanding the performance of these models on standardized tests is crucial for evaluating their potential and limitations [10, 11].

Although studies have provided valuable insights into how commercially available VLMs perform in the Japanese medical field, a comprehensive evaluation of a broader range of models, including state-of-the-art reasoning models and locally run open-source models, is still needed. Furthermore, understanding the strengths and weaknesses of different model architectures and training paradigms in handling different question types, such as image interpretation, regulatory knowledge, and specific organ systems, is essential to assess their potential usefulness in nuclear medicine education and practice [13].

This study aims to address these gaps by evaluating the performance of several state-of-the-art VLMs, including the latest commercially available reasoning models (ChatGPT o1 pro, ChatGPT o1, ChatGPT o3-mini, Claude 3.7, Gemini 2.0 Flash thinking), non-reasoning commercial models (ChatGPT-4.5) and locally run open-source models (Llama3.2, Gemma 3), on the JNMBE questions from 2022 to 2024. By analyzing their accuracy across different question formats, including those with and without images, as well as across various subspecialties and regulatory aspects, this research seeks to provide a comprehensive assessment of the current capabilities of VLMs in the context of nuclear medicine specialization in Japan. The findings of this study will contribute to a better understanding of the potential and limitations of these advanced AI models in supporting nuclear medicine education, training, and potentially clinical practice in the future.

## Materials and method

### Exam questions dataset

We obtained the complete set of questions from the JNMBE for the years 2022, 2023, and 2024. These exams are administered annually by the Japanese Board of Nuclear Medicine, and, after the exam, the questions are made publicly available for educational purposes. We obtained permission from the Education Committee of the Japanese Board of Nuclear Medicine to use the JNMBE questions for this study. Questions and accompanying images are publicly available on the JSNM website after each examination. Each yearly exam consists of 60 multiple-choice questions. The questions cover a range of topics in nuclear medicine, including clinical case diagnosis using imaging, radiopharmaceutical characteristics, instrumentation and physics, image analysis, and relevant regulations. Many questions with images include one or more images (e.g., planar scintigraphy, single-photon emission computed tomography (SPECT) PET images, fused PET/CT images, or schematics of uptake patterns) as part of the question. Out of the 180 total questions across 2022–2024, 58 questions (32%) were identified as questions with images, meaning they required interpreting a provided image in conjunction with the text, while the remaining 122 (68%) were text-only questions. Each question had five answer options with a single correct answer. The official correct answers were not available. No patient-identifiable information was present in any question, and all content was already public; therefore, this study did not require IRB approval. Two board‑certified nuclear‑medicine physicians (RI and YA) prepared answers independently, and in cases where answers diverged, a third board‑certified nuclear‑medicine physician (RM) provided a decision. One nuclear medicine physician (RI) and one radiologist (KK) evaluated whether the questions included images, whether the image questions were in the field of cardiology, neurology, oncology nuclear medicine, or general nuclear medicine, and whether the questions were related to Japanese law according to the Guidelines for the JNMBE.

### LLMs

We selected advanced vision LLMs available as of March 2025, focusing on models that are rated as state-of-the-art or representative in the field. All selected models are capable of processing image inputs along with text. We used six cloud models: ChatGPT o1 Pro, ChatGPT o1, ChatGPT o3-mini, ChatGPT-4.5, Claude 3.7 Sonnet and Gemini 2.0 Flash thinking [1–5]. ChatGPT o1 Pro, ChatGPT o1, ChatGPT o3-mini, ChatGPT-4.5, Claude 3.7 Sonnet, and Gemini 2.0 Flash thinking are reasoning models. Reasoning models are models that have enhanced reasoning capabilities. ChatGPT-4.5 is a non-reasoning model. These models are widely used around the world. We also used two local models: Local Llama 3.2 (70B), Gemma 3 (27B) [6, 7]. These models were run on an on-premises workstation with an NVIDIA RTX A6000 GPU (48 GB VRAM) and NVIDIA RTX 3090 (24 GB VRAM), respectively. These 2 local models are non-reasoning models.

### Image pre‑processing and inference pipeline

All cloud-based and local models received the JPEG files extracted from the original PDF file directly from the user interface at the same time as receiving the question. Images were handled simultaneously, not by prior image data input or post-image input.

### Inference procedure

All inputs were in the original Japanese language of the questions. We used a uniform, simple Japanese prompt for every question: "問題を解いてください。" (Please solve this problem.) If the question included an image, the image (in JPEG format) was provided to the model along with the question text. We did not enforce any specific answer format. To ensure consistency and reduce randomness, the temperature was set to 0 for all local models, ensuring deterministic outputs. In contrast, the cloud models were tested via their web interfaces using default parameters, as the parameters are not public and cannot be changed. These settings can introduce variability in outputs, as parameters like temperature are not open. Because of inherent non-determinism, each question was run three times, with the final answer determined by a majority vote.

### Data analysis

We compared model performance quantitatively. Confidence intervals (95%) for accuracy percentages were calculated using the Wilson method. We used McNemar’s test to compare paired proportions where applicable, comparing a model’s success on image-based questions versus text-only questions, and comparing the head-to-head performance of two models on the same set of questions. A Cochran’s Q test followed by post hoc pairwise McNemar tests (with p-value adjustment) was used to assess differences among all models on the overall accuracy. Performance differences on law-related versus non-law questions were assessed using the Wilcoxon rank-sum test for paired. Year-by-year trends were examined by treating each year’s 60 questions as a block and assessing accuracy changes. Statistical significance was defined as *p* < 0.05. All analyses were performed using R (v4.4.1).

## Results

### Overall accuracy of models

**Fig. 1:**
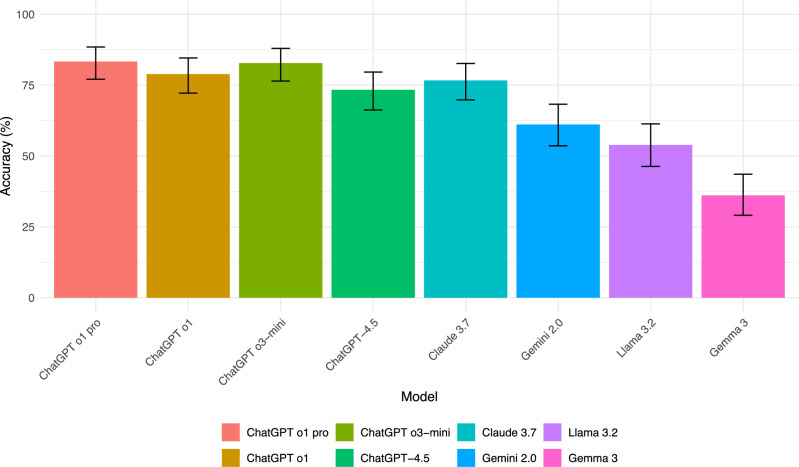
Overall accuracy of VLMs on JNMBE 2022–2024 Each bar represents the overall accuracy (proportion of correct answers out of 180 total questions) for the respective model, with error bars indicating the 95% confidence interval for accuracy. ChatGPT o1 pro achieved the highest accuracy (83.3%), followed closely by ChatGPT o3-mini (82.8%), ChatGPT o1 (78.9%), Claude 3.7 (76.7%), and ChatGPT-4.5 (73.3%). Gemini 2.0 (61.1%), Llama 3.2 (53.9%), and Gemma 3 (36.1%) performed comparatively lower

Figure 1 shows the overall accuracy of each VLM across 180 questions. ChatGPT o1 pro performed best with 83.3% accuracy (150 correct answers), followed closely by ChatGPT o3-mini at 82.8% (149 correct answers). ChatGPT o1 achieved 78.9% accuracy (142 correct), while Claude 3.7 scored 76.7% (138 correct). ChatGPT-4.5 had 73.3% accuracy (132 correct), Gemini 2.0 scored 61.1% (110 correct), Llama 3.2 reached 53.9% (97 correct), and Gemma 3 had the lowest performance at 36.1% (65 correct). Some models (ChatGPT o1 pro, ChatGPT-4.5, Llama 3.2, and Gemma 3) successfully answered all questions based on the majority rule, while others (ChatGPT o3-mini with 2 unanswered, ChatGPT o1 with 8 unanswered, Claude 3.7 with 4 unanswered, Gemini 2.0 with 1 unanswered) either failed to provide an answer consistently across the three runs or gave different answers each time.

A statistical comparison using Cochran's Q test revealed highly significant differences among the models (Q = 235.5, df = 7, *p* = 3.27E-47). Post hoc pairwise McNemar tests (Table 1) identified significant differences between many pairs. Notably, ChatGPT o1 pro significantly outperformed ChatGPT-4.5, Gemini 2.0, Llama 3.2, and Gemma 3 (*p* < 0.01). ChatGPT o3-mini similarly outperformed these lower-ranked models. Gemini 2.0, Llama 3.2, and Gemma 3 showed significantly lower performance compared to all higher-ranked models (*p* < 0.01 generally), with Gemma 3 performing significantly worse than all others. No significant differences were found between the top three (ChatGPT o1 pro, ChatGPT o3-mini, ChatGPT o1) or between ChatGPT o1, ChatGPT o3-mini, Claude 3.7, and ChatGPT-4.5 after p-value adjustment in most pairwise comparisons.

**Table 1:** Pairwise model comparison

Comparison	chi_squared	*p*_value	*p*_adj
ChatGPT o1 pro vs ChatGPT o1	1.56	0.21	1.00
ChatGPT o1 pro vs ChatGPT o3-mini	0.64	0.42	1.00
ChatGPT o1 pro vs ChatGPT-4.5	12.89	0.00	0.01
ChatGPT o1 pro vs Claude 3.7	5.04	0.02	0.69
ChatGPT o1 pro vs Gemini 2.0	29.25	0.00	0.00
ChatGPT o1 pro vs Llama 3.2	38.73	0.00	0.00
ChatGPT o1 pro vs Gemma 3	72.90	0.00	0.00
ChatGPT o1 vs ChatGPT o3-mini	0.08	0.77	1.00
ChatGPT o1 vs ChatGPT-4.5	6.04	0.01	0.39
ChatGPT o1 vs Claude 3.7	0.89	0.34	1.00
ChatGPT o1 vs Gemini 2.0	21.78	0.00	0.00
ChatGPT o1 vs Llama 3.2	30.82	0.00	0.00
ChatGPT o1 vs Gemma 3	68.60	0.00	0.00
ChatGPT o3-mini vs ChatGPT-4.5	7.03	0.01	0.22
ChatGPT o3-mini vs Claude 3.7	2.04	0.15	1.00
ChatGPT o3-mini vs Gemini 2.0	25.52	0.00	0.00
ChatGPT o3-mini vs Llama 3.2	34.91	0.00	0.00
ChatGPT o3-mini vs Gemma 3	72.30	0.00	0.00
ChatGPT-4.5 vs Claude 3.7	1.88	0.17	1.00
ChatGPT-4.5 vs Gemini 2.0	7.52	0.01	0.17
ChatGPT-4.5 vs Llama 3.2	14.02	0.00	0.01
ChatGPT-4.5 vs Gemma 3	48.96	0.00	0.00
Claude 3.7 vs Gemini 2.0	15.85	0.00	0.00
Claude 3.7 vs Llama 3.2	24.45	0.00	0.00
Claude 3.7 vs Gemma 3	62.64	0.00	0.00
Gemini 2.0 vs Llama 3.2	1.62	0.20	1.00
Gemini 2.0 vs Gemma 3	26.27	0.00	0.00
Llama 3.2 vs Gemma 3	15.50	0.00	0.00

We compared model performance on 10 law-related questions versus 170 non-law questions (Table 2). ChatGPT o1 pro showed a statistically significant drop in accuracy on law-related questions (60.0%) compared to non-law questions (84.7%, *p* = 0.043, Wilcoxon rank-sum test). While all other models also performed worse on law questions, the differences were not statistically significant, although the trend was consistent. Accuracy on law questions ranged from 30.0% (Gemma 3) to 70.0% (ChatGPT o1, ChatGPT o3-mini).

**Table 2:** Accuracy (%) on law-related vs. non-law questions

Model	Law related (N = 10)	Non-law (N = 170)	*p*-value
ChatGPT o1 pro	60.0	84.7	0.043
ChatGPT o1	70.0	79.4	0.70
ChatGPT o3-mini	70.0	83.5	0.23
ChatGPT-4.5	60.0	74.1	0.33
Claude 3.7	60.0	77.7	0.38
Gemini2.0	50.0	61.8	0.45
Llama 3.2	40.0	54.7	0.37
Gemma 3	30.0	36.5	0.68

**Fig. 2:**
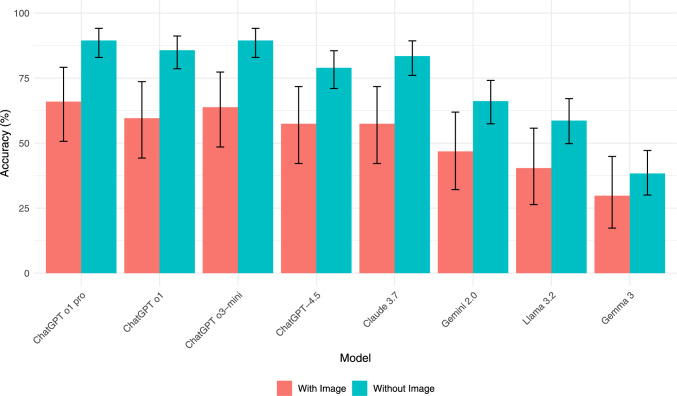
Comparison of eight language models with and without image-based inputs For each model, the orange bar indicates accuracy when questions include images (With Images), and the teal bar indicates accuracy for text-only questions (Without Images). In general, most models demonstrate higher performance on text-only questions, with statistically significant differences (*p* < 0.05) for all but Gemma 3

All models performed substantially better on text-only questions (N = 122) compared to questions requiring image interpretation (N = 58), as shown in Fig. 2. The performance drop was statistically significant for all models except Gemma 3 (*p* < 0.001 for ChatGPT o1 pro, ChatGPT o1, ChatGPT o3-mini, Claude 3.7; *p* < 0.01 for GPT4.5; *p* < 0.05 for Gemini2.0, Llama 3.2; McNemar test). For ChatGPT o1 pro, accuracy fell from 89.5% (text-only) to 66.0% (image). Similar drops were seen for ChatGPT o3-mini (89.5% to 63.8%), ChatGPT o1 (85.7% to 59.6%), and Claude 3.7 (83.5% to 57.4%). Gemma 3 showed the lowest performance in both conditions and no significant difference.

**Fig. 3:**
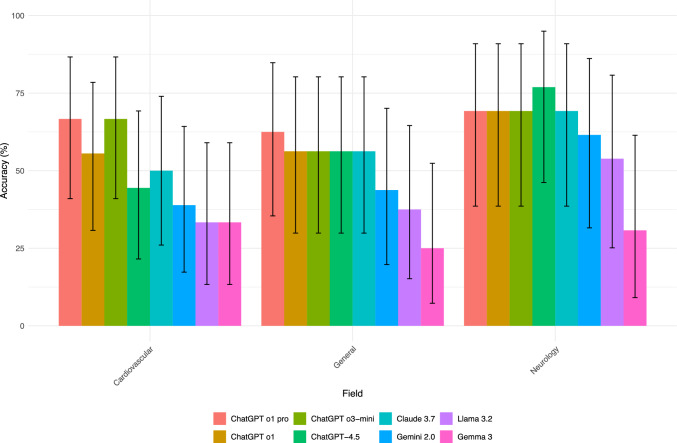
Performance comparison of eight language models across three medical domains (Cardiovascular, General, and Neurology) Bars indicate each model’s accuracy—calculated as the percentage of correct answers out of the total number of questions—with error bars denoting 95% confidence intervals. Each domain is grouped together for a side-by-side view of how the models’ performances vary by specialty. Overall, ChatGPTo1 pro, ChatGPT-4.5, and ChatGPT o3-mini tend to achieve higher accuracy in most domains, whereas models like Gemini 2.0, Llama3.2, and Gemma 3 show comparatively lower performance

Image questions were categorized into cardiology (N = 18), neurology (N = 13), general nuclear medicine, and oncology nuclear medicine (N = 16). Performance varied by domain (Fig. 3). In the Cardiovascular field, ChatGPT o1 pro and ChatGPT o3-mini tied for the highest accuracy at 66.7%, followed by ChatGPT o1 at 55.6%. Claude 3.7 achieved 50% accuracy, while ChatGPT4.5, Gemini2.0, Llama 3.2, and Gemma 3 had lower scores. For General Nuclear medicine questions, ChatGPT o1 pro led with 62.5% accuracy, followed by Chat GPT o1, ChatGPT o3-mini, ChatGPT-4.5, and Claude 3.7 all tied at 56.2%. Gemini2.0, Llama 3.2, and Gemma 3 performed less well. In Neurology, ChatGPT-4.5 showed the strongest performance with 76.9% accuracy, while ChatGPT o1 pro, ChatGPT o1, ChatGPT o3-mini, and Claude 3.7 all tied for second place at 69.2%. Gemini2.0 achieved 61.5%, Llama 3.2 53.8%, and Gemma 3 had the lowest score at 30.8%.

Analysis of performance from 2022 to 2024 (Table 3) shows relative stability for top models but declines for lower-performing ones. ChatGPT o1 pro (85.0, 83.3, 81.7%) and ChatGPT o3-mini (85.0, 83.3, 80.0%) maintained high accuracy. ChatGPT o1 (80.0, 80.0, 76.7%) and Claude 3.7 (75.0, 80.0, 75.0%) were also relatively consistent. ChatGPT-4.5 showed fluctuation (65.0% in 2022, 81.7% in 2023, 73.3% in 2024). Gemini 2.0 remained moderate (60.0, 65.0, 58.3%). Llama 3.2 (56.7 to 45.0%) and Gemma 3 (41.7 to 26.7%) showed downward trends.

**Table 3:** Accuracy (%) by year (95% CI)

Model	2022	2023	2024
ChatGPT o1 pro	85.0 (73.4–92.9)	83.3 (71.5–91.7)	81.7 (69.6–90.5)
ChatGPT o1	80.0 (67.7–89.2)	80.0 (67.7–89.2)	76.7 (64.0–86.6)
ChatGPT o3-mini	85.0 (73.4–92.9)	83.3 (71.5–91.7)	80.0 (67.7–89.2)
ChatGPT-4.5	65.0 (51.6–76.9)	81.7 (69.6–90.5)	73.3 (60.3–83.9)
Claude 3.7	75.0 (62.1–85.3)	80.0 (67.7–89.2)	75.0 (62.1–85.3)
Gemini 2.0	60.0 (46.5–72.4)	65.0 (51.6–76.9)	58.3 (44.9–70.9)
Llama 3.2	56.7 (43.2–69.4)	60.0 (46.5–72.4)	45.0 (32.1–58.4)
Gemma 3	41.7 (29.1–55.1)	40.0 (27.6–53.5)	26.7 (16.1–39.7)

## Discussion

This study evaluated the performance of eight contemporary VLMs on three years (2022–2024) of the JNMBE. Our principal finding is that while top-performing models, ChatGPT o1 pro and o3-mini, achieved high overall accuracy, their performance heavily relied on text-based reasoning. All evaluated models demonstrated a substantial and statistically significant performance drop when faced with questions requiring image interpretation compared to text-only questions. This underscores a critical limitation in the current generation of VLMs regarding specialized medical image analysis. Our findings support the growing consensus that modern VLMs can effectively address textual questions in radiology or nuclear medicine [10, 11, 15, 16].

Our study extends previous evaluations of VLMs in JNMBE conducted in Japan. Oura et al. [15] evaluated VLMs on the Japanese board exams for diagnostic radiology, nuclear medicine, and interventional radiology and reported a range of performances. In the JNMBE (2019–2023), GPT-4o achieved an overall accuracy of 64% (59% for image questions), while other models such as Claude scored lower. In our study, the best model (ChatGPT o1 pro) showed an accuracy of 83.3% and 66% for image questions. Although a simple comparison is not possible, it is possible that accuracy has improved due to model advances. Watanabe et al.[16] evaluated recent models (GPT-4o, Claude 3 Opus, Gemini 1.5 Pro) specifically in the JNMBE (2019–2023) and reported that GPT-4o and Claude 3 Opus had higher accuracy (around 54.3% overall) when provided image questions. In our study, the more recent model (o1pro) had a higher accuracy (66%). Although it is difficult to make a simple evaluation based on these results, it seems that there has been a 20% increase in text problems and a 5–10% increase in image questions over the past year (Table 4). This progress is amazing, but there seems to be a dissociation between the progress of text-based problems and the progress of image problems.

**Table 4:** Comparison with existing LLM research for JNMBE

Study	Exam period	Overall accuracy (%)	Image question accuracy (%)
Oura et al. [15]	2019–2023	64 (GPT-4o)	59 (GPT-4o)
Watanabe et al. [16]	2019–2023		54.3 (GPT-4o, Claude 3)
Our study	2022–2024	83.3 (ChatGPT o1 pro)	66.0 (ChatGPT o1 pro)

The difficulty VLMs encountered with questions pertaining to Japanese regulations (significantly impacting ChatGPT o1 pro's score and trending lower for others) is another key finding. This likely reflects the models' training data, which, while vast, may lack sufficient depth or focus on region-specific legal and regulatory frameworks pertinent to Japanese nuclear medicine practice [13]. General LLMs often struggle with highly specialized, localized knowledge unless specifically fine-tuned or provided with relevant context during inference [10, 11]. This highlights a critical area for improvement if these models are to be considered reliable aid in specific clinical or administrative contexts requiring regulatory adherence. Moreover, fluctuations in examination difficulty from one year to the next could partly account for model‑level performance swings observed in this study.

Interestingly, domain-specific analysis of image questions revealed variations. ChatGPT-4.5's relatively strong performance in neurology image questions (76.9%) warrants further investigation, though the small number of questions per domain limits definitive conclusions. It might suggest biases in training data or specific architectural strengths suited to features in those neurological images. Conversely, the leading models (ChatGPT o1 pro/ChatGPT o3-mini) showed better performance in cardiology and general/oncology nuclear medicine image questions compared to lower-ranked models, suggesting better overall visual feature extraction and reasoning capabilities, though still significantly lagging their text-only performance. These findings should be interpreted with caution. A key limitation of this subgroup analysis is the small number of questions available for each category. Consequently, the performance differences observed may not be statistically robust and should be considered exploratory at this stage.

Year-by-year analysis suggests that the top-performing models are relatively stable. The downward trend observed for the lower performing, locally run models (Llama 3.2, Gemma 3) might reflect inherent limitations due to scale and training paradigms or potential skewing if older examination data were disproportionately represented in their training sets relative to the newest exams.

This study provides a comprehensive evaluation using a relatively large set of official board examination questions over three years. It assesses a diverse range of recent VLMs, including commercial and open-source options. The analysis across different question types, content domains, and examination years provides granular insights. The use of majority voting helps mitigate potential non-determinism.

However, several limitations exist. First, the JNMBE format (multiple-choice) may not fully capture real-world clinical reasoning complexities. Second, the reference standard, though rigorous (physician consensus), is not infallible. Third, AI is evolving rapidly; these benchmarks might be quickly outdated. Fourth, our prompt requested only the final answer choice, so the models' underlying reasoning processes were not captured, making a detailed failure analysis impossible. Furthermore, our study included a mix of architectures, with only some being designated 'reasoning models'. Applying a technique like chain-of-thought (CoT) prompting across this diverse set would have been methodologically inconsistent, as it is not equally suited to all model types. Therefore, while a qualitative analysis of failure modes using CoT is an important research goal, we consider it a distinct follow-up study that should build upon the baseline performance comparison established here. Fifth, we used simple prompts. Because the purpose of this study is to make comparisons, we used the same simple prompts for all models. It is possible that we could get better results by calibrating the prompts to suit each model. Sixth, image-based questions constituted only 32% of the total dataset, and when these were further divided into subdomains like cardiology or neurology, the sample size for each analysis became small. This low number of questions particularly restricts the statistical power of our subdomain performance analysis. Therefore, the observed differences in model accuracy between nuclear fields should be considered preliminary findings that require further validation with larger datasets. Finally, cloud model behavior with default settings introduces potential variability. Future work should explore prompt engineering, model fine-tuning for medical imaging, and integration with explainability methods. It is important to note that our study assessed the models' intrinsic abilities without Retrieval-Augmented Generation (RAG). Future work should explore integrating RAG, which could substantially improve performance. For instance, providing access to a database of regulations could address weaknesses on legal questions, while retrieving similar cases or literature could enhance image interpretation. The integration of RAG represents a critical next step, alongside prompt engineering and fine-tuning, toward developing more reliable clinical and educational AI tools.

Despite these limitations, VLMs hold considerable promise as supplementary tools in medical education and board preparation, an area where they are not meant to replace experts but to support trainees. For instance, trainees could use VLMs as interactive study partners to tackle a large volume of text-based questions, receiving immediate feedback and explanations to reinforce foundational knowledge in areas like radiopharmaceuticals, physics, and clinical guidelines. In a supervised setting, reviewing a VLM's analysis of a case—including its errors in image interpretation—could serve as a valuable educational exercise. This would sharpen a trainee's critical thinking and diagnostic skills by highlighting the nuances that currently distinguish human expertise from artificial intelligence. By integrating VLMs into training curricula in this manner, educators can augment traditional teaching methods, providing scalable and accessible resources to prepare the next generation of nuclear medicine specialists.

## Conclusion

This evaluation of contemporary vision-language models (VLMs) on the Japanese Nuclear Medicine Board Examination revealed that while ChatGPT o1 pro and ChatGPT o3-mini achieved high overall accuracy (83.3 and 82.8%, respectively), all models demonstrated significant performance drops when handling image-interpretation questions compared to text-only questions, with most models also struggling with Japanese regulation-specific content; these findings suggest that while current VLMs can serve as valuable educational tools for reinforcing nuclear medicine knowledge, their substantial limitations in specialized medical image interpretation and regulatory understanding make them suitable only as supplementary aids rather than replacements for expert human judgment or comprehensive training programs.

## Ethics approval

Because this study only used publicly available data, approval by the Nagoya University Ethics Committee was deemed unnecessary.

## Data Availability

The Japanese Nuclear Medicine Board Examination datasets were open access. The analysis data generated during the current study are available from the corresponding author on reasonable request.
